# The Total Urine Protein-to-Creatinine Ratio Can Predict the Presence of Microalbuminuria

**DOI:** 10.1371/journal.pone.0091067

**Published:** 2014-03-10

**Authors:** Kyoko Yamamoto, Hiroyuki Yamamoto, Katsumi Yoshida, Koichiro Niwa, Yutaro Nishi, Atsushi Mizuno, Masanari Kuwabara, Taku Asano, Kunihiro Sakoda, Hiroyuki Niinuma, Fumiko Nakahara, Kyoko Takeda, Chiyohiko Shindoh, Yasuhiro Komatsu

**Affiliations:** 1 Department of Laboratory Medicine and Clinical Science, Tohoku University Graduate School of Medicine, Sendai, Japan; 2 Division of Nephrology, Department of Internal Medicine, St. Luke's International Hospital, Tokyo, Japan; 3 Healthy Medical Center, Tohoku Kosai Hospital, Sendai, Japan; 4 Department of Cardiology, Cardiovascular Center, St. Luke's International Hospital, Tokyo, Japan; 5 Clinical Laboratory Department, St. Luke's International Hospital, Tokyo, Japan; University of Tokushima, Japan

## Abstract

**Background:**

The Kidney Disease: Improving Global Outcomes chronic kidney disease (CKD) guidelines recommend that CKD be classified based on the etiology, glomerular filtration rate (GFR) and degree of albuminuria. The present study aimed to establish a method that predicts the presence of microalbuminuria by measuring the total urine protein-to-creatinine ratio (TPCR) in patients with cardiovascular disease (CVD) risk factors.

**Methods and Results:**

We obtained urine samples from 1,033 patients who visited the cardiovascular clinic at St. Luke's International Hospital from February 2012 to August 2012. We measured the TPCR and the urine albumin-to-creatinine ratio (ACR) from random spot urine samples. We performed correlation, receiver operating characteristic (ROC) curve, sensitivity, and subgroup analyses. There was a strong positive correlation between the TPCR and ACR (R^2^ = 0.861, p<0.001). A ROC curve analysis for the TPCR revealed a sensitivity of 94.4%, a specificity of 86.1%, and an area under the curve of 0.903 for detecting microalbuminuria for a TPCR cut-off value of 84 mg/g of creatinine. The subgroup analysis indicated that the cut-off value could be used for patients with CVD risk factors.

**Conclusions:**

These results suggest that the TPCR with an appropriate cut-off value could be used to screen for the presence of microalbuminuria in patients with CVD risk factors. This simple, inexpensive measurement has broader applications, leading to earlier intervention and public benefit.

## Introduction

Microalbuminuria is an early sign of progressive cardiovascular and renal disease in individuals with certain conditions, such as hypertension, diabetes mellitus, and cardiovascular disease (CVD) [1], [2]. Furthermore, a reduction in urinary albumin excretion after treatment with either an angiotensin-converting enzyme inhibitor or angiotensin II receptor blocker is associated with good long-term effects with regard to cardiovascular mortality [3]. The estimated glomerular filtration rate (eGFR) and albuminuria are independently associated with all-cause mortality and cardiovascular mortality [4]. The American Heart Association recommends combined screening tests for a low eGFR and microalbuminuria in patients with CVD risk factors to assess chronic kidney disease (CKD) risk [5], [6].

The Kidney Disease: Improving Global Outcomes (KDIGO) CKD guidelines recommend that CKD should be classified based on the cause, glomerular filtration rate and degree of albuminuria [7]. Although microalbuminuria is preferred as a marker over the total urine protein-to-creatinine ratio (TPCR), the cost of measuring albumin may limit its use in some countries [8]–[11].

In our previous study in a diabetic population, we reported that there was a significant positive correlation between the TPCR and the urine albumin-to-creatinine ratio (ACR) (r = 0.95) and that the TPCR could predict the presence of microalbuminuria in more than 90% of diabetic patients [12]. However, the relationship between the ACR and TPCR has not been studied in nondiabetic individuals, because measuring microalbuminuria is reimbursed only for diabetic patients in Japan. The upper limit of normal for urine total protein excretion for adults is generally 150 to 200 mg/day [13], [14]. The Japanese Society of Nephrology and the KDIGO CKD guidelines [15] state that a TPCR of 150 mg/g of creatinine (15 mg/mmol) is equal to an ACR of 30 mg/g of creatinine (3 mg/mmol).

In our study, we aimed to examine the utility of the TPCR in predicting the presence of microalbuminuria in patients with CVD risk factors with or without diabetes and determine the appropriate cut-off value for microalbuminuria.

## Subjects and Methods

### Study design

This investigation was a cross-sectional study.

### Patients

**Figure 1:**
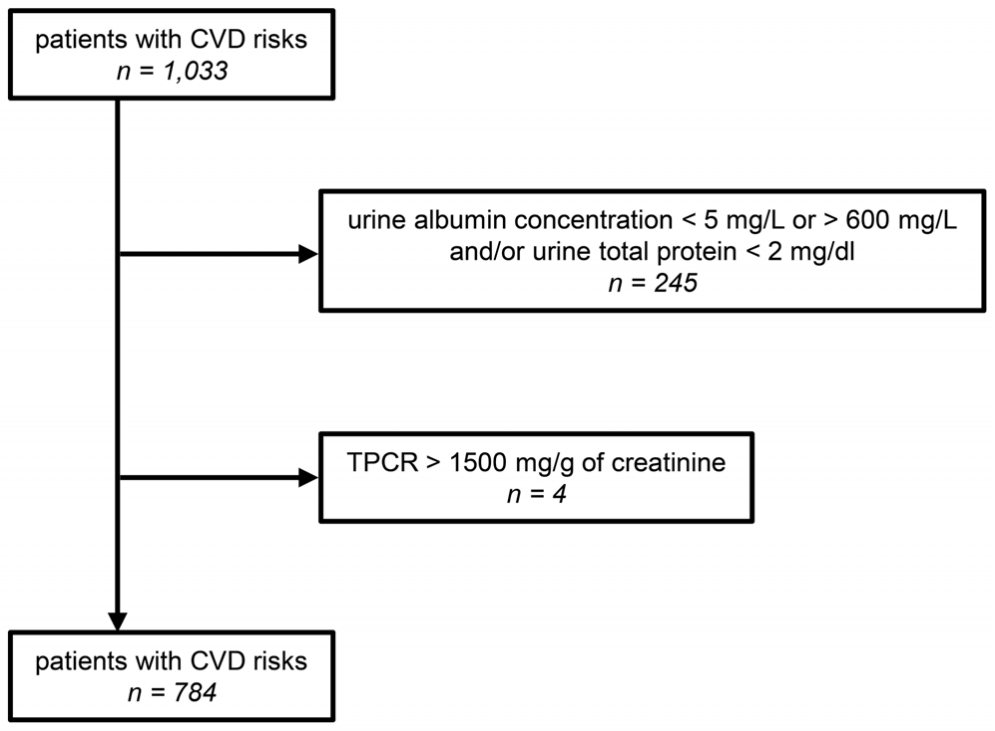
Inclusion criteria. Of the 1,033 adult patients from the cardiovascular clinic at St. Luke's International Hospital, 249 patients who presented with out-of-measurement range or the TPCR>1500 mg/g of creatinine (150 mg/mmol) were excluded from this study. CVD, cardiovascular disease; TPCR, total urine protein-to-creatinine ratio.

For this study, we obtained random spot urine samples from 1,033 consecutive adult patients who visited the outpatient clinic of the Department of Cardiology, Cardiovascular Center at St. Luke's International Hospital in Tokyo from February 1, 2012 to August 31, 2012, and we performed a series of blood and urine tests. Of these 1,033 patients, we excluded 245 patients who had a urine albumin level less than 5 mg/L or greater than 600 mg/L, and/or a urine protein level less than 2 mg/dl, because these levels were outside of the measurement ranges. In addition, we excluded four patients with a TPCR greater than 1500 mg/g of creatinine (150 mg/mmol). Thus, we included a total of 784 patients [age, 69±12 (mean ± SD) years; 280 females, 504 males] in this study (Figure 1).

This study was approved by the Research Ethics Committee at St. Luke's International Hospital in Tokyo. All data used for the current study were collected from patients' medical record including blood and urine data. Blood and urine laboratory studies were performed as a part of standard medical practice. Waiver of informed consent was approved by the Research Ethics Committee.

### Methods

We conducted a semiquantitative dipstick analysis of the urine protein concentration using reagent strips (Eiken Chemical, Tochigi, Japan). The urine was considered to be positive for protein when the dipstick test result was “1+” to “2+” and negative when the dipstick test result was “−” to “+−” (trace). We placed these 784 patients into four groups according to the urine protein results from the dipstick test: individuals with “−” were placed in Group (−) (n = 525 patients); “+−” patients were in Group (+−) (n = 148 patients); “1+” patients were assigned to Group (1+) (n = 91 patients); and “2+” patients were placed in Group (2+) (n = 20 patients). On the same day, we measured the total urine protein, albumin, and creatinine levels in the urine and serum low-density lipoprotein (LDL) cholesterol, C-reactive protein (CRP), and creatinine in each sample. We quantified the urine total protein concentration using a pyrogallol red molybdate assay (Micro TP-AR; Wako Pure Industrial, Osaka, Japan) and found that the inter-assay coefficient of variation (CV) for a concentration of 9.4 mg/dl was 0.8%. The urine albumin concentration was measured by immunonephelometry (Kanto Chemical, Tokyo, Japan), and the CV of an 11.5-mg/L concentration was 1.3%. The creatinine concentration was measured enzymatically (KAINOS Laboratories, Inc., Tokyo, Japan), and the CV of a 0.90-mg/dl concentration was 0.7%. The TPCR and ACR were expressed as milligrams per gram (mg/g). Microalbuminuria was defined as an ACR of 30–300 mg/g of creatinine (3–30 mg/mmol) and macroalbuminuria as an ACR over 300 mg/g of creatinine (>30 mg/mmol), respectively. Normoalbuminuria was defined as an ACR<30 mg/g of creatinine (3 mg/mmol) [16]. The estimated GFR was calculated using the formula shown below, which has been adapted for Japanese individuals and is recommended by the Japanese Society of Nephrology [17]: eGFR (mL/min/1.73 m^2^)  = 194× serum creatinine^−1.094^ (mg/dl)× age^−0.287^ (years)× 0.739 (if female).

Hospital laboratory quality for measuring urine total protein, urine creatinine, and dipstick urine protein analysis was assured by the annual external quality assessment conducted by the Japanese Association of Medical Technologists. We obtained the following data from the electronic medical record systems in the hospital: main diagnoses, age at the time of the laboratory tests, gender, blood pressure, rate of albuminuria, and the use of an angiotensin-converting enzyme inhibitor (ACEi), angiotensin receptor blocker (ARB), aldosterone blocker (AldB), direct renin inhibitor (DRI) or statin. A diagnosis of diabetes mellitus was defined as taking diabetes medication or having a hemoglobin A1C of 6.5% or greater.

### Statistics

The data are expressed as the mean ± standard deviation for normal distributions and the median (interquartile range) for non-normal distributions. The relationship between the ACR and TPCR was examined using regression analysis following a log transformation of the values due to the non-normal distribution. We used receiver operating characteristic (ROC) curve analysis to identify the optimal TPCR cut-off value for predicting microalbuminuria. For the sensitivity analysis, we estimated the sensitivity, specificity and ROC area (including 95% confidence intervals) for different cut-off points for comparison with our obtained cut-off value. For the subgroup analysis, we assessed the obtained cut-off value for the TPCR to predict microalbuminuria according to age, gender, the presence or absence of diabetes mellitus, blood pressure, CRP, urine creatinine level [18], [19], eGFR, and the use of drugs (ACEi/ARB/AldB/DRI and statin), and estimated the sensitivity, specificity and ROC area (including 95% confidence intervals) for each group. In the subgroup analysis, only the patients with missing values were excluded from each group. All of the statistical tests were performed using Stata version 12 (Stata Corporation, College Station, TX) and JMP version 9 (SAS, Cary, NC). A two-sided p<0.05 was considered to be significant.

## Results

A total of 784 patients were evaluated in this study. Table 1 shows the main diagnoses. The most frequent clinical diagnosis was ischemic heart disease (n = 322, 41.1%), followed by hypertension (n = 140, 17.9%), arrhythmia (n = 103, 13.1%) and heart failure (n = 97, 12.4%).The baseline characteristics for the 784 patients included in the study are listed in Table 2. Albuminuria (n = 288, 36.7%) included both microalbuminuria (n = 256, 32.7%) and macroalbuminuria (n = 32, 4.1%). Of the 784 patients, 215 (27.4%) patients had diabetes mellitus. Of the 215 diabetic patients, 109 (50.7%) patients had albuminuria; whereas of the 569 non-diabetic patients, 179 (31.5%) patients had albuminuria.

**Table 1:** Main diagnoses for patients at the cardiovascular clinic.

Main Diagnoses	Number	Percent (%)
Ischemic heart disease	322	41.1
Hypertension	140	17.9
Arrhythmia	103	13.1
Heart failure	97	12.4
Valvular disease	34	4.3
Aortic dissection/aneurysm	30	3.8
Cardiomyopathy	23	2.9
Arteriosclerosis obliterans	9	1.1
Congenital heart disease	9	1.1
Pulmonary embolism	7	0.9
Other	10	1.3
Total	784	100

**Table 2:** Baseline patient characteristics.

Characteristics	784 patients	Missing value (%)
Age (years)	69±12 (range = 29–98)	0
Male (%)	504 (64.3)	0
Albuminuria (%)	288 (36.7)	0
Microalbuminuria (%)	256 (32.7)	0
Macroalbuminuria (%)	32 (4.1)	0
Diabetes mellitus (%)	215 (27.4)	0
Systolic blood pressure (mmHg)	129±11	15.6
Diastolic blood pressure (mmHg)	76±7	15.6
Laboratory test		
LDL cholesterol (mg/dl)	103.7±27.9	0.5
C-reactive protein (mg/dl)	0.06 (IQR 0–0.14)	2.4
Serum creatinine (mg/dl)	0.79 (IQR 0.65–0.95)	0
eGFR (mL/min/1.73 m^2^)	69.7±20.5	0
TPCR (mg/g of creatinine)	73.2 (IQR 44.4–135.8)	0
ACR (mg/g of creatinine)	18.9 (IQR 10.1–51.8)	0
Medications		
ACEi and/or ARB (%)	486 (62.0)	0
Aldosterone blocker (%)	71 (9.1)	0
Direct renin inhibitor (%)	12 (1.5)	0
Statin (%)	365 (46.6)	0

The data are presented as the mean ± standard deviation or as the median (interquartile range), as appropriate. Albuminuria includes both micro- and macroalbuminuria. LDL, low-density lipoprotein; eGFR, estimated glomerular filtration rate; TPCR, total urine protein-to-creatinine ratio; ACR, urine albumin-to-creatinine ratio; IQR, interquartile range; ACEi, angiotensin-converting enzyme inhibitor; ARB, angiotensin receptor blocker.

### Albuminuria and urine protein using a dipstick test

The urine protein level was negative [Groups (−) and (+−)] in 673 patients. Of these patients, 126 patients (18.7%) in Group (−) and 64 patients (9.5%) in Group (+−) had albuminuria. Furthermore, 11.7% of patients with proteinuria [Groups (1+) and (2+)] were negative for albuminuria. The proportion of patients with albuminuria increased as the urine protein level by the dipstick test increased (Table 3).

**Table 3:** Albuminuria and urine protein by dipstick test.

Dipstick test	Albuminuria (%)			Total (%)
	Normo-	Micro-	Macro-	
−	399 (76.0)	126 (24.0)	0	525 (67.0)
+−	84 (56.8)	61 (41.2)	3 (2.0)	148 (18.9)
1+	11 (12.1)	63 (69.2)	17 (18.7)	91 (11.6)
2+	2 (10.0)	6 (30.0)	12 (60.0)	20 (2.6)
Total (%)	496 (63.3)	256 (32.7)	32 (4.1)	784 (100)

### Correlation between the ACR and TPCR

**Figure 2:**
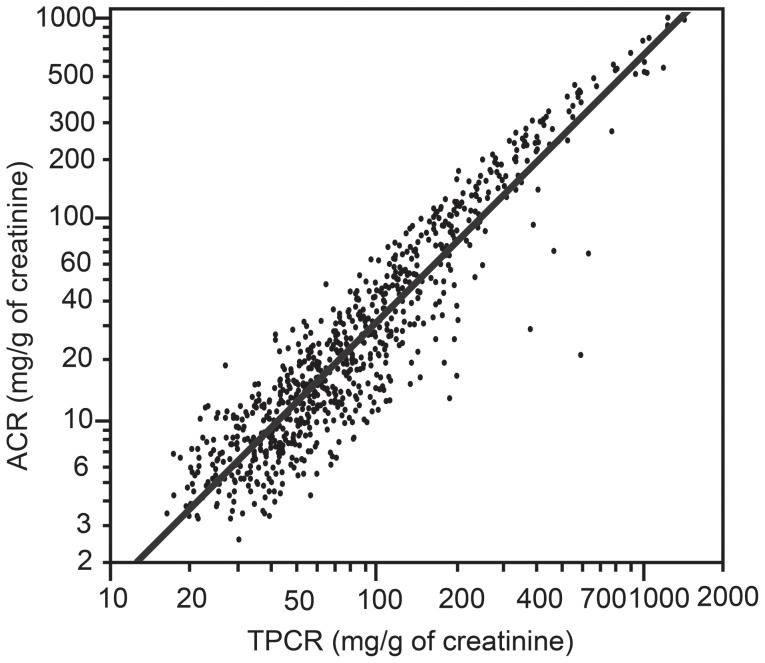
Correlation between the urine albumin-to-creatinine ratio (ACR) and the total urine protein-to-creatinine ratio (TPCR). All of the axes are on a logarithmic scale. The regression expression was written as follows: ln ACR = 1.32×ln TPCR – 2.64 [coefficient of determination R^2^ = 0.861 (p<0.001)].

**Figure 3:**
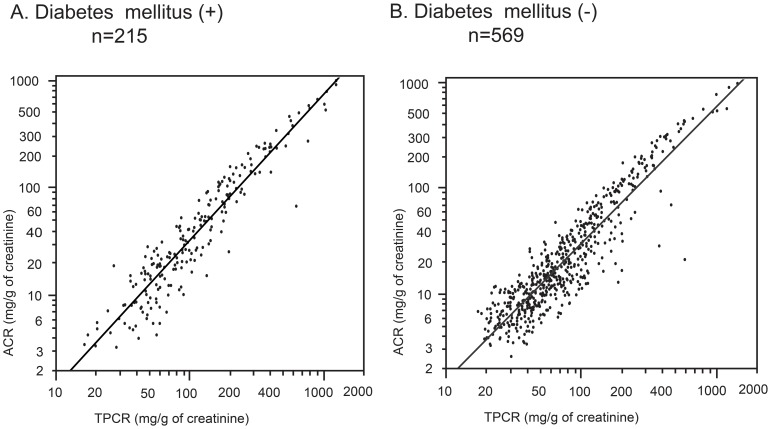
Correlation between the urine albumin-to-creatinine ratio (ACR) and the total urine protein-to-creatinine ratio (TPCR) in the patients with or without diabetes mellitus. All of the axes are on a logarithmic scale.

There was a strong positive correlation between the ACR and TPCR. The regression expression was written as follows: ln ACR = 1.32×ln TPCR −2.64 [coefficient of determination R^2^ = 0.861 (*p*<0.001)] (Figure 2). The correlations between ACR and TPCR in patients with or without diabetes were comparable with the results observed for the patient population (Figure 3).

### ROC curve analysis and sensitivity analysis

**Figure 4:**
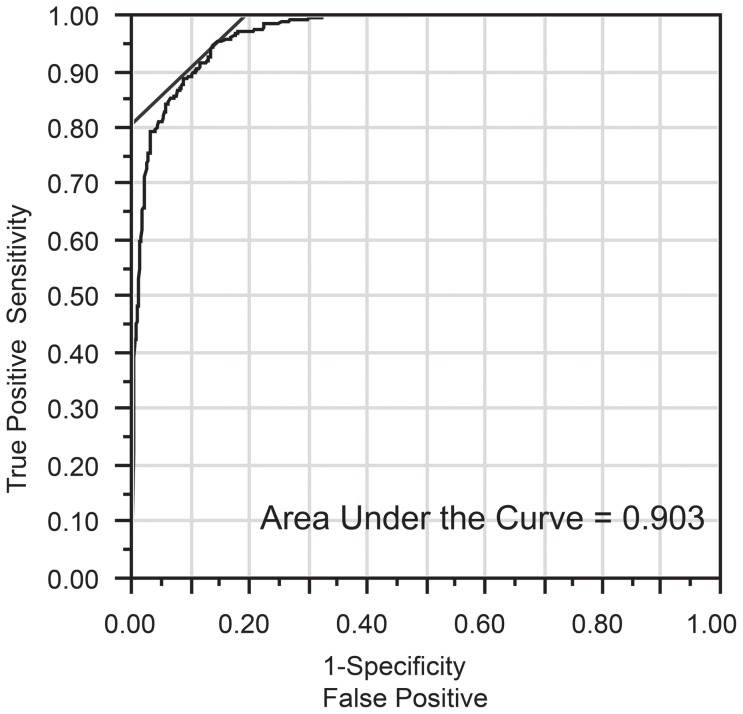
Receiver operating characteristic curve analysis for the total urine protein-to-creatinine ratio (TPCR) and albuminuria. The optimal cut-off value for the TPCR to predict microalbuminuria was 84 mg/g of creatinine (8.4 mg/mmol).

**Figure 5:**
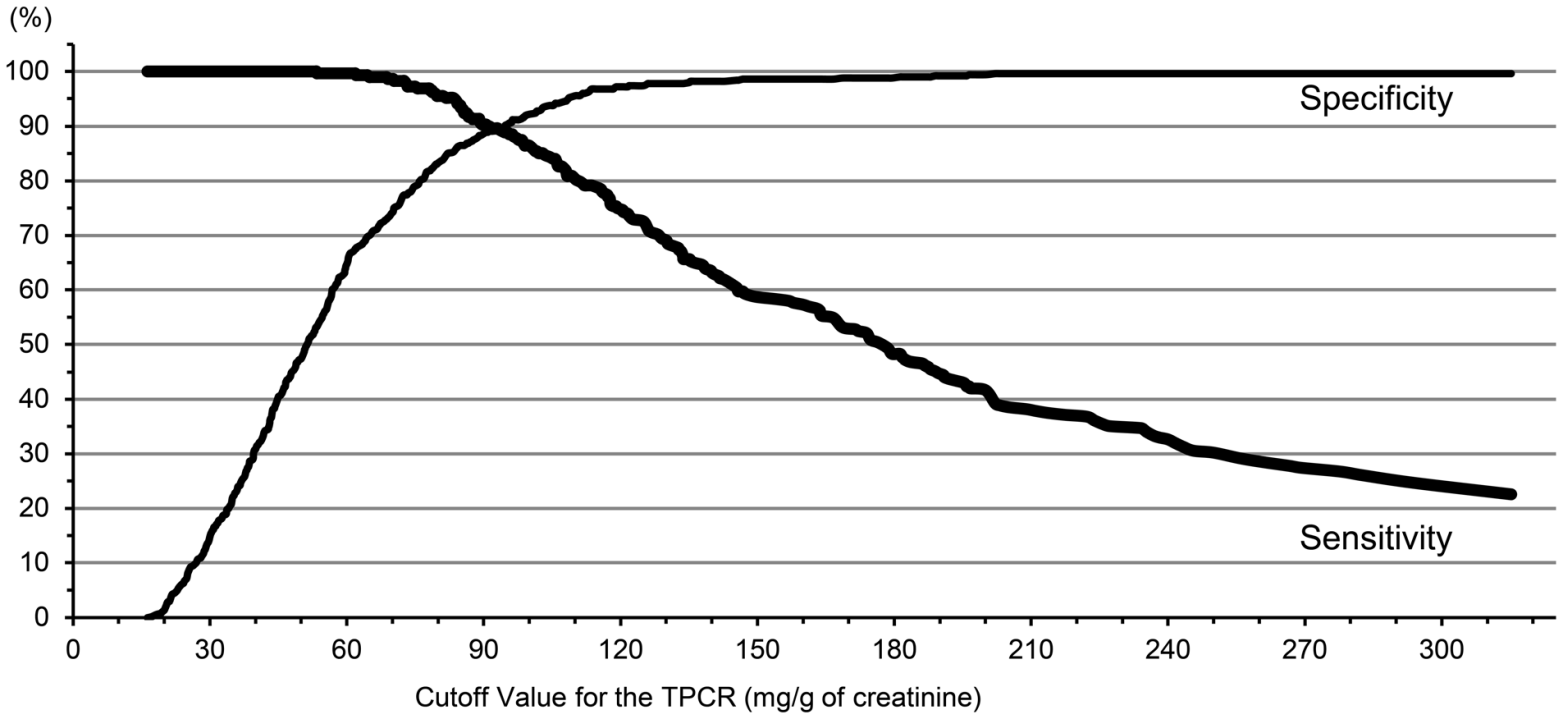
Relationship between the cut-off value and specificity/sensitivity. TPCR, total urine protein-to-creatinine ratio. Note that the higher cut-off value increased the specificity while decreasing the sensitivity.

The optimal cut-off value for the TPCR based on the ROC curve analysis for predicting microalbuminuria was 84 mg/g of creatinine (8.4 mg/mmol) (Figure 4). The sensitivity, specificity, and AUC were 94.4%, 86.1%, and 0.903, respectively, for this cut-off value (Table 4). There were 85 discordant results concerning the ACRs and TPCRs (false negative in 16 cases and false positive in 69 cases). By altering the cut-off value from 80 mg/g of creatinine (8.0 mg/mmol) to 90 mg/g of creatinine (9.0 mg/mmol), the sensitivity decreased, and the specificity increased. There was no significant difference in the ROC area among the three groups. At a cut-off value of 150 mg/g of creatinine (15.0 mg/mmol), the sensitivity was very low (58.7%), and the ROC area (0.786) was significantly decreased (Table 4, Figure 5).

**Table 4:** Sensitivity analysis of the cut-off value.

Cut-off Value (mg/g of creatinine) [mg/mmol]	Sn (%)	Sp (%)	ROC area [95% CI]
80 [8.0]	95.5	83.7	0.896 [0.876–0.916]
*84 [8.4]	94.4	86.1	0.903 [0.882–0.923]
90 [9.0]	90.3	88.9	0.896 [0.874–0.918]
150 [15.0]	58.7	98.6	0.786 [0.757–0.815]

Sn, sensitivity; Sp, specificity; CI, confidence interval; ROC, receiver operating characteristic.

*84 mg/g of creatinine (8.4 mg/mmol) for the TPCR was the optimal cut-off value to predict microalbuminuria based on the receiver operating characteristic curve analysis.

### Subgroup analysis

**Figure 6:**
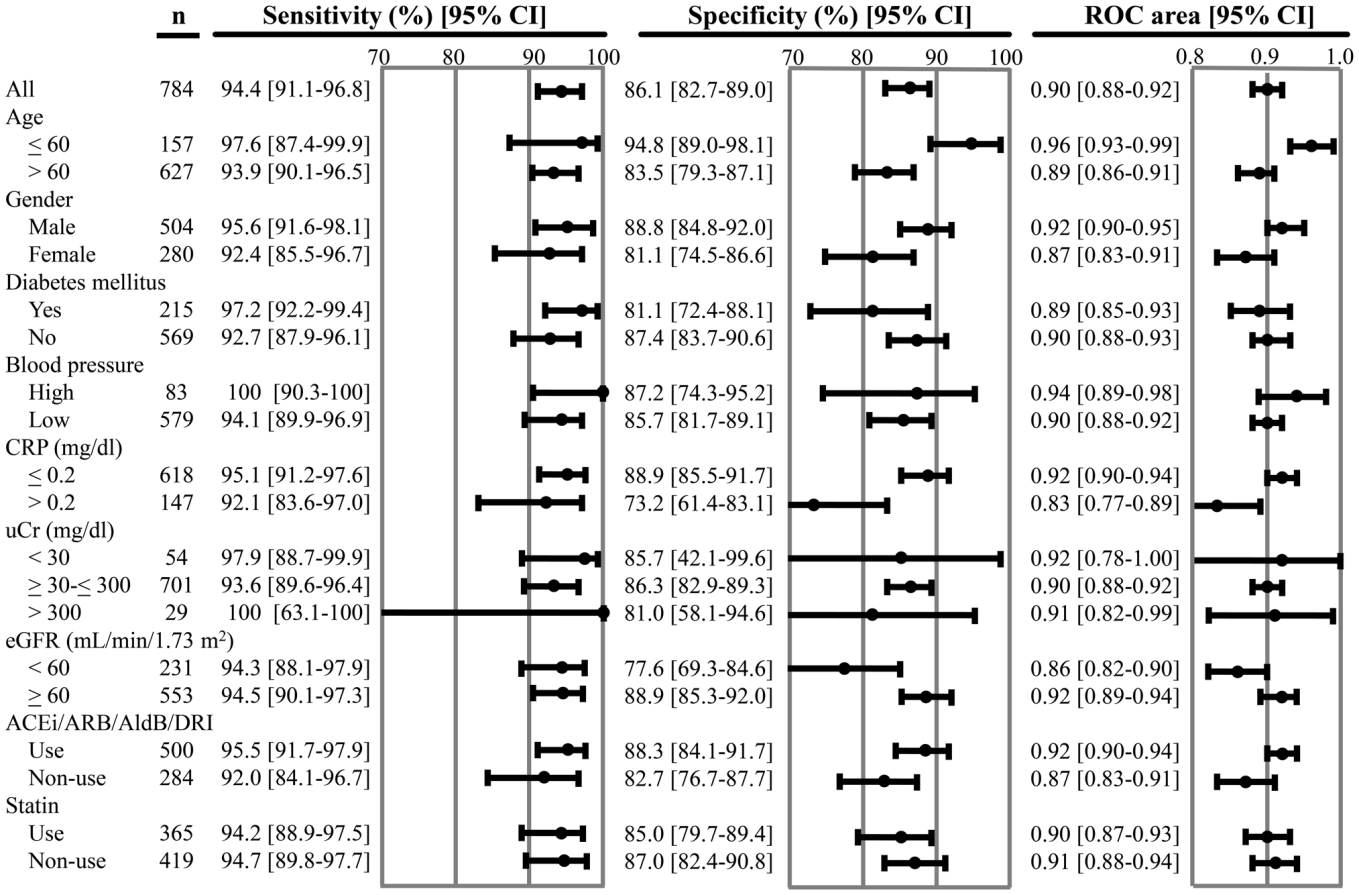
Subgroup analysis. All, all patients combined; CRP, C-reactive protein; uCr, urine creatinine; eGFR, estimated glomerular filtration rate (ml/min/1.73 m^2^); ACEi, angiotensin-converting enzyme inhibitor; ARB, angiotensin receptor blocker; AldB, aldosterone blocker; DRI, direct renin inhibitor; ROC, receiver operating characteristic; CI, confidence interval. Age is expressed in years. High blood pressure was defined as a systolic blood pressure >140 mmHg or diastolic blood pressure >90 mmHg.

The subgroup analysis was performed to examine the effect of the various factors on predicting microalbuminuria; the factors studied were age, gender, the presence or absence of diabetes mellitus, blood pressure, CRP, urine creatinine level, eGFR, and the use of drugs (ACEi/ARB/AldB/DRI and statin). The subgroup analysis indicated that a cut-off value of 84 mg/g of creatinine (8.4 mg/mmol) could be generally applicable to patients with CVD risk factors (Figure 6).

Regarding sensitivity, none of these variables had a significant effect at a cut-off value of 84 mg/g of creatinine (8.4 mg/mmol) for the TPCR to predict microalbuminuria. However, several variables had a significant effect on specificity. The specificity was significantly higher for age≤60 years compared to >60 years, those with a CRP≤0.2 compared to >0.2, and those with an eGFR ≥60 compared to <60. However, no significant difference was observed if these groups were compared to a group combining all of the patients. Similarly, the ROC area was significantly higher for age ≤60 years compared to >60 years and those with a CRP ≤0.2 compared to >0.2. When these groups were compared to the combined group, only those younger than 60 years were significantly different.

## Discussion

The present study showed that there was a strong positive correlation between the ACR and TPCR and the presence of microalbuminuria could be predicted by determining TPCR in patients with CVD risk factors with or without diabetes. Several studies have reported the relationship between the ACR and TPCR [20], [21]. However, they did not examine whether measuring the TPCR can predict the presence of microalbuminuria. Our previous study reported, for the first time, that the presence of microalbuminuria could be predicted by measuring the TPCR in patients with diabetes mellitus [12].

In the present study, the cut-off value obtained from the ROC curve analysis, which was selected to maximize the AUC, indicated that the optimal TPCR cut-off value for predicting microalbuminuria was 84 mg/g of creatinine (8.4 mg/mmol). This cut-off value had a sufficient sensitivity, specificity, and AUC (94.4%, 86.1%, and 0.903, respectively). These results were comparable to the findings in our previous study [12]. We have already reported that the optimal cut-off value is 91 mg/g of creatinine (9.1 mg/mmol) in diabetic patients. In the sensitivity analysis, when the cut-off value was changed from 80 mg/g of creatinine (8.0 mg/mmol) to 90 mg/g of creatinine (9.0 mg/mmol), the sensitivity decreased, the specificity increased, and there was no significant difference in the ROC area. Using a cut-off value of either 80 mg/g of creatinine (8.0 mg/mmol) or 90 mg/g of creatinine (9.0 mg/mmol) would be acceptable to predict microalbuminuria using the TPCR in patients with CVD risk factors. The selection of a cut-off value requires a trade-off between sensitivity and specificity.

The upper limit of normal for urine total protein excretion is 150 to 200 mg/day for adults [13], [14]. Most nephrologists use this cut-off for discerning pathologic from physiologic proteinuria. The Japanese Society of Nephrology and the KDIGO CKD guidelines [15] have shown that a TPCR of 150 mg/g of creatinine is equivalent to microalbuminuria with 30 mg/g of creatinine; however, the evidence is limited. In this study, a cut-off value of 150 mg/g of creatinine had a remarkably lower sensitivity, higher specificity and significantly lower ROC area compared to a cut-off value of 84 mg/g of creatinine. If we use 150 mg/g of creatinine as a cut-off value, approximately 40% (119/288) of microalbuminuria-positive patients were not detected, representing false negatives. In contrast, if we use a TPCR of 84 mg/g of creatinine as the cut-off value, the percentage of false-negative patients was only 2% (16/784), and we can detect most of the microalbuminuria-positive patients. Considering these results, a cut-off value of 84 mg/g of creatinine would be superior to the more commonly used cut-off of 150 mg/g of creatinine, at least for the patients with CVD risk factors.

In our subgroup analysis, the value of 84 mg/g of creatinine (8.4 mg/mmol) could be applicable to patients with CVD risk factors. The specificity was significantly lower in those with a CRP>0.2 and eGFR<60 than in those with a CRP≤0.2 and eGFR≥60. Therefore, the number of false positives may be increased in these groups but would not pose any clinical problem, considering the similar specificities in the range of 95% confidence interval for all of the patients. Our subgroup analysis demonstrated that the specificity was significantly higher in the ≤60 years of age, CRP≤0.2, and eGFR≥60 groups. These results favor this cut-off in a screening program, targeting younger patients and those with normal renal function.

One of the barriers to conducting albuminuria screening in the clinical setting is the cost of measuring urine albumin, which is expensive compared to measuring total protein. The reagent costs for measuring the ACR and TPCR are £2.16 and £1.42, respectively, in the United Kingdom [8], whereas these costs are £0.4/€0.5 and £0.15/€0.2 in Australia [9], respectively. In Canada, the laboratory analysis costs (Canadian dollars) are $2.81 for reagent strips, $29.23 for the ACR and $11.67 for the TPCR [10]. In the United States, reimbursements for measuring the ACR and TPCR are $16 and $13, respectively [11]. In Japan, reimbursements by the National Health Insurance for measuring the ACR and TPCR are ¥1,130 and ¥70, respectively. In Japan, the cost of measuring the ACR is more than 10-fold higher than that of the TPCR. Furthermore, in Japan, the national health insurance only reimburses ACR measurement for diabetic patients; individuals with a high risk for cardiovascular disease are not reimbursed unless they have diabetes. Alternatively, measuring the TPCR is simple and inexpensive, thus permitting its widespread use for screening. Recent studies on general Dutch population have reported that population-based screening for albuminuria might be cost-effective [22], [23]. However, to demonstrate the advantage of TPCR, further studies based on cost-effectiveness trials in clinical settings would be required.

Our study has some limitations. First, this study is a single-center, hospital-based study. Second, some patients were excluded because the levels of urine albumin and/or urine protein were outside the measurement ranges in this study. Most of these excluded patients had extremely low levels of urine albumin and/or urine protein. To establish the use of the TPCR in clinical practice, additional strategies, such as the use of a morning urine sample, which should be highly concentrated, will be required. Third, the results can be applied to patients with CVD risk factors. To test the applicability of our study for general population screening, a multicenter, population-based study is required.

In conclusion, the present study shows that the TPCR can predict the presence of microalbuminuria and can be used as an inexpensive method to screen for microalbuminuria in patients with CVD risk factors. We can use the TPCR to diagnose the presence and degree of microalbuminuria in clinical practice and to conduct follow-up studies on these patients. In an era of increasing healthcare costs, the use of the TPCR may result in equivalent outcomes at a reduced medical cost.
